# Sleep Disordered Breathing in Hypertrophic Cardiomyopathy—Current State and Future Directions

**DOI:** 10.3390/jcm9040901

**Published:** 2020-03-25

**Authors:** Shreyas Venkataraman, Shahid Karim, Aiswarya Rajendran, C. Anwar A. Chahal, Virend K. Somers

**Affiliations:** 1 Department of Cardiovascular Medicine, Mayo Clinic, Rochester, MN 55905, USA; Venkataraman.shreyas@mayo.edu (S.V.); Karim.shahid@mayo.edu (S.K.); Rajendran.aiswarya@mayo.edu (A.R.); Anwar.Chahal@pennmedicine.upenn.edu (C.A.A.C.); 2 Philadelphia Heart Institute, University of Pennsylvania School of Medicine, Philadelphia, PA 19104, USA; 3 Division of Cardiology, Royal Papworth Hospital, Cambridge CB2 0AY, UK

**Keywords:** Sleep apnea, hypertrophic cardiomyopathy, sleep-disordered breathing

## Abstract

Hypertrophic cardiomyopathy (HCM) is the most common inherited cardiomyopathy and sleep disordered breathing (SDB) is a treatable risk factor that has been seen to occur concurrently, and is known to propagate mortality and morbidity in a number of cardiovascular disease states including heart failure, and indeed hypertrophic cardiomyopathy. In this review, we summarize past studies that explored the simultaneous occurrence of HCM and SDB, and the pathophysiology of SDB in relation to heart failure, arrhythmias, cardiac ischemia and pulmonary hypertension in HCM. The current therapeutic modalities, with the effect of obstructive sleep apnea (OSA) treatment on HCM, are then discussed along with potential future directions.

## 1. Introduction

Hypertrophic cardiomyopathy (HCM) has been a known cause of cardiac morbidity and mortality with the first pathological report documented in the 1950s by Teare [1] at St. George’s Hospital, London. Following this, Braunwald and colleagues described the clinical profile of the condition [2], and over the ensuing years various names like idiopathic hypertrophic subaortic stenosis (IHSS) and hypertrophic obstructive cardiomyopathy (HOCM) were coined, describing what was believed to be the hallmark features of obstruction [3]. HCM is presently recognized as the commonest inherited cardiac condition, where population studies have estimated the prevalence of HCM in the population to be at least one in 500 [4]. Contemporary guidelines define HCM as a condition characterized by unexplained left ventricular (LV) hypertrophy that is not explained by abnormal loading conditions and occurs in the absence of another cardiac or systemic disease that would be capable of producing the magnitude of hypertrophy seen in a patient [3]. In the clinical setting, HCM is said to be highly likely in the presence of asymmetric LV wall thickening (≥15 mm on echocardiography) [3]. The majority of adult HCM is due to sarcomeric gene mutations, inherited in an autosomal dominant fashion, with variable penetrance and clinical heterogeneity. However, 5–10% of adult cases are caused by various other genetic conditions including hereditary neuromuscular and metabolic diseases, chromosomal abnormalities and genetic syndromes, which may have different degrees of skeletal muscle involvement. In pediatric populations, sarcomeric causes dominate, but non-sarcomeric causes are more frequent than in adults [5], and can be associated with more severe disease and earlier expression [6].

An apnea is the absence of inspiratory airflow for at least 10 s. Hypopnea on the other hand, is a reduction in airflow lasting 10 s or longer, which is associated with a fall in arterial oxyhemoglobin saturation or an arousal. Obstructive sleep apnea (OSA) is an acquired clinical condition, defined as a ≥10 s pause in respiration associated with ongoing respiratory effort. A diagnosis is made when a patient has an apnea–hypopnea index (AHI: Number of apneas and hypopneas per hour of sleep) of >5 with increased daytime sleepiness [7]. Snoring and wake-time sleepiness are the most common manifestations of OSA; however, although they are relatively sensitive, they lack specificity [8]. 

OSA is a significant reversible cause of many cardiovascular diseases including hypertension [9], myocardial infarction [10], atrial fibrillation (AF) [11,12], sudden cardiac death (SCD) [13] and renal disease [14]. Most importantly, it is known to be associated with an increased arrhythmia risk in both normal populations and in populations with established cardiovascular diseases such as ischemic heart disease and heart failure [15,16]. There exist a number of mechanisms by which morbidity and mortality in hypertrophic cardiomyopathy may be advanced by the presence of sleep apnea, as has been demonstrated in heart failure patients. Given the limited advances in treatment of hypertrophic cardiomyopathy over the past two decades, this is an important potential avenue for treatment and warrants further exploration. 

Here, we review the literature on OSA and its role in HCM patients, with an emphasis on symptoms, heart failure, outflow tract obstruction, AF, ventricular arrhythmias (VA) and SCD. We also seek to identify potential challenges and opportunities in understanding the role OSA plays in adult HCM patients, along with its future directions in investigation and management.

## 2. Review Criteria

To explore all studies looking at the association between sleep disordered breathing (SDB) and HCM, a search for articles published between 1st January 1980 and 1st November 2019 were included in the MEDLINE database. The following key words were used: sleep-disordered breathing, obstructive sleep apnea, central sleep apnea, cardiomyopathy and hypertrophic cardiomyopathy. All studies were carefully examined for methodology used in recruitment, diagnosis and assessment of cardiovascular outcomes in HCM patients with OSA. 

## 3. Prevalence of Sleep Disordered Breathing (SDB) in Hypertrophic Cardiomyopathy (HCM)

An increased prevalence of SDB (as defined in Appendix A.) has been observed in both adult and pediatric populations. Among the pediatric population, a pilot study [17] with overnight polysomnography (PSG) showed that 48% of 21 patients had SDB. Among these, 24% had central sleep apnea (CSA) while the remaining had OSA.

Among adults, the first evidence of increased prevalence of sleep apnea in HCM was published by Banno et al. [18], where among patients with different cardiomyopathies, 15 patients with HCM were studied. Overnight PSG done in these patients showed seven (47%) patients to have OSA; no patient was found to have CSA. 

**Figure 1:**
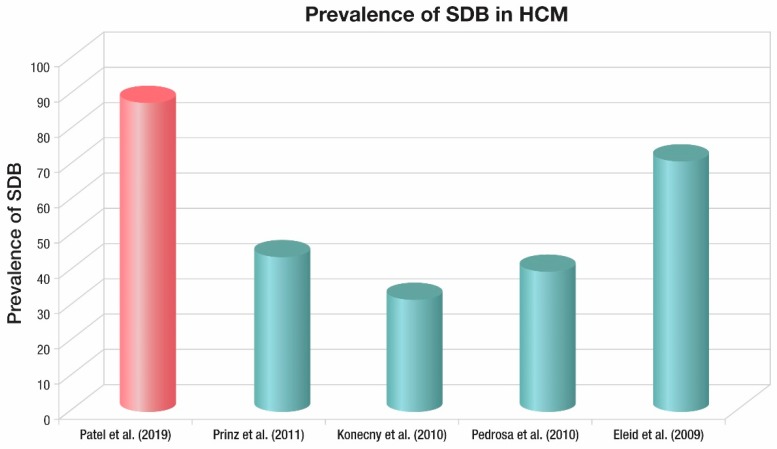
Summary of Prevalence of SDB in HCM.

Following this, as in Figure 1, four observational studies published between 2009 and 2011 showed the prevalence of SDB to range from 32% to 71% [19,20,21,22]. Although the prevalence of SDB was clearly high in these studies, these studies utilized overnight oximetry or a type III portable monitor to diagnose SDB. These studies are not as sensitive and specific in diagnosing and classifying SDB as overnight PSG, and overnight oximetry cannot reliably distinguish between central and obstructive forms.

Additionally, as shown in Table 1, the majority of patients in all four studies were largely male, elderly and had relatively higher body mass indices (BMI). This may reflect a selection bias as well as changes in temporal trends. Patients referred for overnight oximetry or PSG were likely selected on the basis of clinical symptoms, body habitus and pre-test probabilities, thus demonstrating a higher diagnostic yield. These studies therefore provided estimated frequencies and not prevalence or incidence given non-representative biased sample sizes, without controls.

As recently as 2019, Patel et al. [23] did a retrospective study where they looked at 94 patients with HCM who underwent clinical PSGs and found 80% of patients to have OSA, 6.6% to have a combination of OSA and CSA and 1.1% to have CSA alone. A major shortcoming of the study was the presence of a referral bias where there was a high pretest probability for SDB and thus these patients were selectively sent for PSG.

## 4. Cardiovascular Diseases and Obstructive Sleep Apnea (OSA)

### 4.1. Pathophysiology

**Figure 2:**
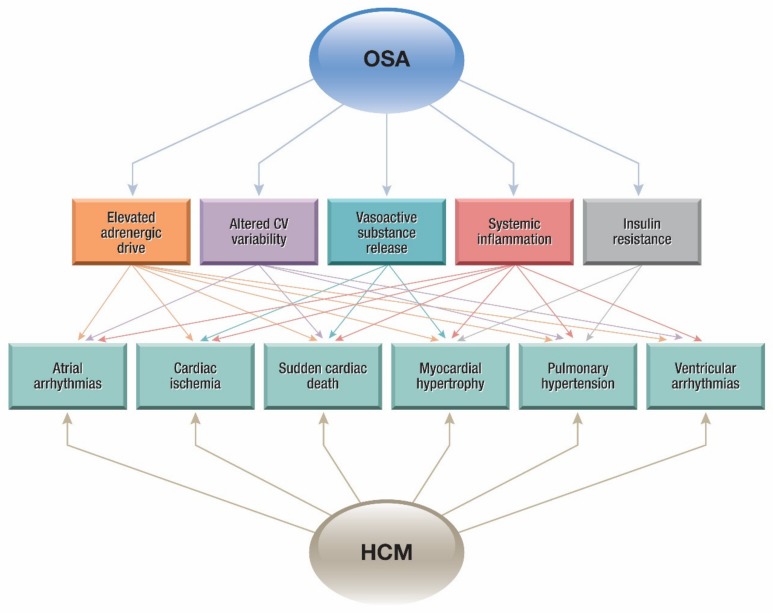
Pathophysiological interaction between obstructive sleep apnea (OSA) and HCM.

There is increasing evidence suggesting that OSA increases the likelihood of cardiovascular risk factors to which HCM patients are uniquely susceptible. As shown in Figure 2, pathophysiological mechanisms implicated include: (1) increased sympathetic activity, driven mainly by recurrent apneas during sleep [24]; (2) altered cardiovascular variability, with OSA patients having decreased heart rate variability and an elevated blood pressure variability [25]; (3) vasoactive substance release, precipitated by hypoxemic stress [26]; (4) triggering of systemic inflammation [27]; (5) insulin resistance and glucose intolerance independent of BMI [28].

OSA is known to increase the risk of factors that contribute to morbidity and mortality in HCM including arrhythmias, myocardial hypertrophy and sudden cardiac death [29]. As shown in Figure 2, factors that contribute to the pathophysiology of OSA are also strongly relevant to the natural history of HCM. Additionally, there is increasing evidence that OSA may increase cardiovascular risk in non-HCM cardiovascular conditions such as heart failure and coronary artery disease, and there have been suggestions to introduce screening for SDB in HCM patients to mitigate this risk [30]. More recent studies provide support for implementing these practices on the bedside. The following section reviews key relationships between OSA and cardiovascular diseases relevant to HCM. 

#### 4.1.1. Heart Failure

Wang et al. [31] showed that of the 164 heart failure patients (but not HCM) studied with overnight PSG, 26% had an AHI ≥ 15 and three months of continuous positive airway pressure (CPAP) attenuated abnormalities in diastolic function. It was observed that nocturnal oxygen desaturation also independently predicted dysfunctional ventricular relaxation during diastole [32].

In HCM, most patients manifest an HFpEF phenotype, while others develop HFrEF at a later stage [3]. HCM patients may have a high prevalence of heart failure, where one study [33], among a cohort of 1000 patients with a confirmed diagnosis of HCM, showed a nearly 50% prevalence of heart failure with mild to severe symptoms. Another study [34], with a larger cohort of 3208 patients, showed a heart failure prevalence of 67%. OSA can also cause the progression of heart failure through multiple mechanisms [14,29] including: (1) increased adrenergic outflow to kidney, heart and vessels; (2) acute and chronic increase in afterload; and (3) increased right ventricular afterload. Progression of heart failure in HCM patients is usually due to severe LV obstruction or adverse LV remodeling, which are both exacerbated by OSA. 

There is evidence to suggest that the increased negative intrathoracic pressure seen in OSA increases transmural pressures across the ventricles, atria and aorta, which could further aggravate LV remodeling and worsen LV obstruction [35]. Furthermore, tissue Doppler imaging studies with Dobutamine stress echocardiography have suggested that OSA may reduce myocardial contractile reserve [36].

#### 4.1.2. Coronary Artery Disease 

Histopathological analysis at post-mortem in HCM patients with SCD demonstrates extensive evidence of myocardial damage [37]. Evidence of all phases of both acute (coagulative necrosis and neutrophilic infiltrate) and subacute (myocytolysis and granulation tissue healing) myocardial ischemia with septal fibrotic scar presence were found in 74% of cases and did not correlate topographically with a major coronary artery. No patients demonstrated significant major epicardial atherosclerotic coronary artery stenosis. However, four out of 19 patients had deep myocardial bridging of the left anterior descending artery.

A number of potential mechanisms have been suggested for microvascular dysfunction in HCM including decreased arteriolar density, fibrosis, myofibril disarray and elevated LV end-diastolic pressure (LVEDP) [38,39]. Reduced diastolic compliance secondary to progressive hypertrophy and disarray increasing stiffness may lead to reduced coronary micro-circulation filling [40]. Myocardial bridging with coronary artery compression may also be implicated.

Abnormal intramural coronary arteries with severely thickened walls and marked luminal narrowing have been found at rates of 20 times more frequently in HCM patients at autopsy than in controls [41]. Intimal hyper-proliferation alongside medial hypertrophy and subsequent luminal narrowing may further ischemia. All of the above, paired with excess myocardial oxygen demand caused by significant LV hypertrophy, may explain the presence of acute as well as chronic changes of ischemia.

A single center cohort study assessed myocardial blood flow (MBF) via positron emission tomography (PET) scan in 51 HCM patients (of whom 14 complained of typical anginal symptoms) once normal coronary arteries had been confirmed via invasive coronary angiography and compared this with 12 control patients complaining of atypical chest pain [42]. MBF was found to be severely reduced in HCM patients as compared to controls. 

The degree of impairment was found to be similar in the intraventricular septum as well as LV free wall. There was no significant difference in MBF based on the presence or absence of anginal symptoms nor left ventricular outflow tract (LVOT) obstruction. Those patients with the most severely reduced MBF were most likely to have progressed to NYHA class III/IV symptoms or require ICD implantation due to development of life-threatening ventricular arrhythmia or to have died from cardiovascular causes after follow-up (average 8.1 years). Severely reduced MBF was later found to be a strong predictor (94% negative predictive value) of progression of adverse ventricular remodeling and systolic impairment even in those who had normal LV cavity sizes and function at initial evaluation [43]. Although the role of SDB on coronary perfusion has been well studied [10,44,45], there are few studies that determine the effect SDB on MBF in HCM. 

#### 4.1.3. Arrhythmias

Atrial Fibrillation:

Atrial fibrillation (AF) is the most common sustained arrhythmia in HCM, approximately 25% of HCM patients will develop AF [46]. While left ventricular filling rates remain unchanged in HCM, the degree of left ventricular filling occurring passively is reduced due to non-compliance. Left ventricular diastolic filling is maintained through increased atrial systolic contribution (31% in HCM patients vs 16% in healthy subjects). This explains why AF is poorly tolerated in HCM. A faster heart rate associated with AF will also lead to reduced diastolic filling time [47]. Higher left atrial pressures and ongoing structural changes correlate with the increasing incidence of AF. Increased atrial interstitial fibrosis is a hallmark of atrial remodeling and a cause of electrical disruption, leading to delayed intra-atrial and inter-atrial conduction [48].

In a porcine model, repeated negative tracheal pressure induction during tracheal occlusion reproducibly shortened atrial refractory period length and significantly increased AF inducibility as well as premature atrial contraction, considered precursors to onset of paroxysms of AF. Vagotomy, atropine or beta blocker administration all influenced said changes, suggesting that these were secondary to sympatho-vagal imbalance [12,49]. When exposed to isolated hypercapnia, a sheep model demonstrated prolongation of the atrial effective refractory period (AERP). While this reduced the likelihood of AF during the period of hypercapnia itself, this was significantly elevated during the period of return to eucapnia [11]. Isolated hypoxia in a rabbit model has been seen to reduce conduction velocity and prolong the refractory period whist reducing homogeneity in conduction as well as inducing pulmonary vein potentials predisposing to atrial arrhythmias including AF [50,51].

Intrathoracic pressure swings caused by intermittent collapse of the airway in OSA induce changes in cardiac transmural pressure and myocardial stretch, particularly affecting the thin-walled atria [52]. Induced negative intrathoracic pressures during repeated Mueller maneuvers (attempted inspiration against a closed glottis) have demonstrated similar changes in atrial dimensions [53].

Interestingly, however, a temporal link between SDB and paroxysms of AF has been demonstrated, suggesting that chronicity and atrial remodeling may not be the only factors inducing OSA-related AF, but also that acute apneic changes may be to blame. These could include blood gas changes, intrathoracic pressure changes or sympatho-vagal imbalance. Indeed, direct recordings of sympathetic nerve activity alongside respiration and blood pressure have demonstrated high levels of resting sympathetic activity during wakefulness and normoxia in OSA. During periods of apnea, hypoxemia and hypercapnia induce further increases in sympathetic activity and surges in systolic blood pressure, both of which were seen to normalize with CPAP treatment [54,55].

Ventricular Arrhythmias:

Hypertrophic areas of ventricular myocardium in HCM display fibrotic changes with significant myocyte disarray. These areas show abnormal electrical conduction with reduced voltage potentials. Acute and sub-acute myocardial ischemia and the associated necrotic changes and those associated with sleep disordered breathing predispose to arrhythmogenesis of which mechanisms include abnormal automaticity with spontaneous electrical impulse formation and propagation, triggered automaticity related to hypoxemia or acidemia, as well as re-entrant circuits [56]. 

A recent study by Wang et al. [57] investigated the association between non-sustained ventricular tachycardia (NSVT) and OSA in HCM patients, as well as echocardiographic differences between HCM patients with and without OSA. A total of 130 HCM patients underwent polysomnographic examination as well as holter monitoring for 24 h and echocardiography. A higher prevalence of NSVT and supraventricular tachycardia (SVT) was seen to be associated with those HCM patients diagnosed with OSA. HCM patients with OSA of increased severity were seen to have a higher prevalence of NSVT as well as SVT.

HCM patients without OSA had a prevalence of NSVT of 12% and SVT of 21% as compared to those with severe OSA (AHI > 30) with a prevalence of NSVT of 54% and SVT of 54%. Interestingly, multivariate analysis demonstrated only family history of HCM or of sudden cardiac death to be independent factors for NSVT with age and left atrial diameter being independent factors for SVT.

In the setting of HCM and its associated subendocardial ischemia, tachycardia driven by excessive sympathetic activation brought about by sleep apnea may further exacerbate ischemia, potentiating the substrate for arrhythmia [57] Certainly, the presence of NSVT has been confirmed as an established risk factor for SCD in HCM as well as for the risk of receiving appropriate shock therapy in HCM patients with a defibrillator in situ [58,59].

#### 4.1.4. Pulmonary Hypertension

Pulmonary hypertension (PH) is characterized by a mean pulmonary artery pressure ≥25 mmHg [60]. PH may develop in patients with HCM, where diastolic dysfunction and mitral regurgitation (secondary to LVOT obstruction) can cause high diastolic pressure in the left side of the heart. Wu et al. [61] showed that HCM patients have a PH incidence of 12.3%. Following this, Musumeci et al. [62] retrospectively analyzed 361 diagnosed HCM patients with echocardiography where they observed 41 patients (11.4%) with PH diagnosed with an elevated pulmonary artery systolic pressure (PASP). They also showed that PH was associated with an unfavorable HCM-related morbidity and mortality risk

PH in HCM is found to be more commonly post-capillary PH than precapillary PH due to an increased left atrial pressure [63]. Sleep apnea could have a contributory role as OSA has been linked to increased left atrial size in HCM, particularly in obstructive HCM [19]. Ong et al. [64] studied a cohort of 1570 patients with HCM who were followed for 3.3 years and observed that a third of these patients had concomitant PH and this was associated with an increased mortality risk. Regarding treatment of OSA, Nerbass et al. [65] examined the role of nasal CPAP in HCM patients and reported that obstructive HCM patients have an elevation in pulmonary artery pressure when on CPAP. These data require further exploration.

## 5. Treatment

### 5.1. Current Treatment Modalities for HCM

The ACCF/AHA hypertrophic cardiomyopathy guidelines [3] state that the most common presentations are: (1) atrial fibrillation, where it is associated with greater risk of stroke and systemic thromboembolism; (2) heart failure, which may be progressive and is characterized by exertional dyspnea; this may progress to end stage with LV remodeling and systolic dysfunction; and (3) SCD due to ventricular arrhythmias.

### 5.2. Prevention of SCD

Initial and periodic (every 12 to 24 months) SCD risk stratification is recommended, where personal history of arrhythmias, family history of SCD (including ICD therapy for ventricular arrhythmias), unexplained syncope, NSVT, left atrial diameter and LV wall thickness ≥30 mm are considered. The HCM risk SCD [66] is a risk prediction model for HCM that had been used to direct the use of implantable cardioverter defibrillators (ICDs) for the prevention of SCD. The risk prediction has been extensively tested for validation and shown to be the best available risk predictor [67,68]. As of now, OSA is not part of the ESC risk calculator, but it could become a part of the prediction model in the future when studies could potentially show evidence of OSA increasing SCD risk.

Other risk modifiers that are considered are: (1) late-gadolinium enhancement (LGE) on cardiac MRIs [69], (2) double mutations with compound heterozygotes and digenic carriers [70], (3) marked LVOT obstruction (Gradient ≥ 30 mmHg), and (4) LV apical aneurysms with LGE [71]. 

### 5.3. Therapy

The guidelines recommend (Class I) that asymptomatic patients be treated for their conditions that may contribute to cardiovascular morbidity (e.g., hypertension, diabetes, hyperlipidemia and obesity). In symptomatic patients, the guidelines suggest both pharmacologic and invasive measures.

Class I pharmacological measures include beta-blocker medications for the treatment of symptoms like angina or dyspnea, where if low doses are ineffective, the dose is titrated to a heart rate (resting) of less than 60–65 bpm. The use of verapamil is recommended for patients who do not respond to beta blocking drugs. IV phenylephrine is recommended for the treatment of obstructive HCM patients with acute hypotension unresponsive to fluid administration. 

Other (Class IIa and IIb) recommendations include adding disopyramide, diltiazem and diuretics for symptoms in patients who do not respond to Class I therapies. Invasive therapies like surgical myectomy or alcohol ablation are recommended for the treatment of eligible patients with severe drug-refractory symptoms and LVOT obstruction.

### 5.4. Effects of OSA Treatment on HCM

CPAP therapy remains the most commonly used and gold standard therapy for OSA. Peker et al. reported a significantly elevated risk of developing of cardiovascular diseases in patients with a history of OSA in the absence of hypertension or cardiovascular disease at baseline with a significant reduction in CVD incidence (OR 0.1) with effective treatment of OSA [72]. Milleron et al. reported significant lower rates of major adverse cardiovascular events (MACE) (defined as a composite of death, myocardial ischemia, acute exacerbation of heart failure or the need for coronary revascularization) in patients with a history of significant CAD and OSA treated with CPAP therapy versus those that were not [73].

When studying 26 patients with HCM—both obstructive and non-obstructive—and the acute effects of 10cm H_2_O CPAP administration vs sham CPAP, the earlier referenced Nerbass study found no changes in blood pressure, cardiac output/stroke volume, ejection fraction or LVOT gradient. Right atrial size was reduced; however, the right ventricular outflow acceleration time was significantly lower, presumably because of an increase in pulmonary artery pressure. Interestingly, left atrial size was acutely reduced in obstructive HCM patients [65]. Indeed, recurrent negative intra-thoracic pressures, hypoxia, and hypercarbia, as well as resultant acidosis, may potentiate pulmonary hypertension.

Altered adrenergic signaling has been reported in both sleep apnea as well as HCM. This has been suggested as one potential mechanism for remodeling seen in HCM; OSA-related hypoxemia—which corrects with CPAP therapy—has also been implicated in the pathogenesis of fibrosis and progression of HCM with OSA [74]. Early data from our ongoing prospective study showed an increased 24-h urine norepinephrine level. 

Systemic blood pressure and sympathetic activity are improved with CPAP therapy, and there is evidence that CPAP therapy may in fact reverse or slow progression of left ventricular hypertrophy [75]. Hence, there is strong precedent to support further research on the downstream effects of treatment of OSA in HCM patients.

## 6. Future Direction

Sleep apnea has a high prevalence in the general population and is a recognized independent risk factor for hypertension, CAD, AF, VA, myocardial infarction, HF and SCD. Observational studies and our ongoing prospective research demonstrate a high frequency of SDB in HCM, a condition associated with a higher than expected frequency of angina, HF syndrome, AF, VA and SCD. The two conditions occurring together pose a particularly unique and exciting challenge in that they may potentiate altered cardiovascular hemodynamics: HCM with obstruction resulting in intra-cavity gradients, diastolic dysfunction, atrial remodeling and an atrial cardiomyopathy and OSA inducing left atrial stretch, changes in intrathoracic pressure affecting venous return and altered neural circulatory control with increased sympathetic drive. Investigating the structural and physiological changes in HCM *with and without* sleep apnea using state-of-the-art imaging and physiological assessment affords the opportunity to investigate and define pathophysiology in a hitherto relatively unexplored area. How OSA interacts with more recent risk modifiers, such as late gadolinium enhancement, awaits further investigation. Most important, however, is whether effective treatment of OSA may improve outcomes in HCM, a question that awaits the initiation and completion of randomized controlled trials. 

## Figures and Tables

**Table 1:** Summary of all published studies assessing sleep disordered breathing in patients with hypertrophic cardiomyopathy (HCM). Baseline characteristics are included.

Author	Prinz et al.	Konecny et al.	Pedrosa et al.	Eleid et al.
**Year published**	2011	2010	2010	2009
**Patients (n)**	63	91	80	100
**HCM patients n (%)**	63 (100)	91 (100)	80 (100)	100 (100)
**Age (years)**	59 (34–85)	52 (20–83)	47 (32–58)	55 (44–75)
**Male (%)**	63	68	49	59
**OSA n (%)**	44	32	40	71
**BMI mean (range) (kg/m^2^)**	26.9 (21.4–32.4)	31 (26–36)	26.4 (17–35.8)	31.1 (24.6–37.6)
**Method used**	Portable monitor	Overnight oximetry	Portable monitor	Overnight oximetry
**Criteria used**	American Academy of Sleep Medicine 1999 criteria	>5 events/hr of decrease in O2 saturation of at least 4%, with a threshold of 90%	American Academy of Sleep Medicine 1999 criteria	>5 events/hr of decrease in O2 saturation of at least 4%, with a threshold of 90%
**RDI/ODI (events/h)**	34.8 (2.3–67.3)	8.6	9.2 (4.1–24.8)	N/A
**Nadir SpO_2_ (%)**	N/A	N/A	84 (78–88)	N/A

**HCM**-Hypertrophic cardiomyopathy; **OSA**-Obstructive sleep apnea; **BMI**-Body mass index; **RDI**-Respiratory disturbance index; **ODI**-Oxygen desaturation index.

## Appendix A

Supplement 1 Definitions. Sleep disordered breathing (SDB): Defined according to the American Academy of Sleep Medicine (AASM) criteria where an apnea is the absence of inspiratory airflow for at least 10s. Hypopnea on the other hand, is a decrease in airflow lasting 10s or longer, which is associated with a drop in arterial oxyhemoglobin saturation or an arousal.
